# Older Adults’ Experiences and Perceptions of Immersive Virtual Reality: Systematic Review and Thematic Synthesis

**DOI:** 10.2196/35802

**Published:** 2022-12-06

**Authors:** David Healy, Aisling Flynn, Owen Conlan, Jenny McSharry, Jane Walsh

**Affiliations:** 1 School of Psychology University of Galway Galway Ireland; 2 School of Nursing and Midwifery University of Galway Galway Ireland; 3 School of Computer Science and Statistics Trinity College Dublin Dublin Ireland

**Keywords:** older adults, virtual reality, immersive virtual reality, aging, systematic review, qualitative evidence synthesis, thematic synthesis

## Abstract

**Background:**

Immersive virtual reality (IVR) can be defined as a fully computer-generated environment shown on a head-mounted display. Existing research suggests that key features of IVR can assist older adults in their everyday lives, providing opportunities for health promotion and tackling social isolation and loneliness. There has been a surge in qualitative studies exploring older adults’ experiences and perceptions of IVR. However, there has been no systematic synthesis of these studies to inform the design of new, more accessible IVR technologies.

**Objective:**

This study aimed to systematically review and synthesize qualitative studies exploring older adults’ experiences and perceptions of IVR.

**Methods:**

A systematic review and thematic synthesis were conducted following the ENTREQ (Enhancing Transparency in Reporting the Synthesis of Qualitative Research) guidelines. In total, 2 reviewers completed title and abstract screening, full-text screening, data extraction, and quality appraisal. Thematic synthesis is derived from the qualitative method, thematic analysis. It involves 3 key steps: initial coding and grouping of these codes, the formation of descriptive themes from these codes, and going beyond these data to form analytical themes. Confidence in the evidence was assessed using the Grading of Recommendations Assessment, Development, and Evaluation-Confidence in the Evidence from Reviews of Qualitative Research approach.

**Results:**

Overall, 13 studies were included in the final synthesis, including 224 participants across 9 countries and 5 continents. Confidence in the evidence ranged from high to moderate. Three descriptive themes were generated: practical aspects of IVR use, experiencing unique features of IVR, and perceptions of IVR. The findings from the descriptive themes suggested that there are several improvements that need to be made to existing IVR devices to facilitate older adults’ use of this technology. However, older adults’ responses to IVR were generally positive. Three analytical themes were generated: tolerating the bad to experience the good, buying in to IVR (don’t judge a book by its cover), and “it proves to me I can do it.” The analytical themes illustrated that older adults were willing to tolerate discomforts that accompany existing IVR technologies to experience features such as immersive social networking. There was a discrepancy between older adults’ perceptions of IVR before use—which were generally negative—and after use—which were generally positive—and IVR provided a platform for older adults to access certain activities and environments more easily than in the real world because of limitations caused by aging.

**Conclusions:**

This review offers insights into older adults’ experiences and perceptions of IVR and suggests how a few improvements to its existing hardware and software as well as how it is first presented could offer new opportunities for older adults to take part in meaningful activities tailored to their needs and preferences.

**Trial Registration:**

PROSPERO CRD42020200774; https://tinyurl.com/8f48w2vt

**International Registered Report Identifier (IRRID):**

RR2-10.1177/16094069211009682

## Introduction

### Rationale

On the basis of projections reported by the World Health Organization (WHO), the number of people aged >60 years will rise from 900 million (12% of the global population) in 2015 to 2 billion (20% of the global population) by 2050 [1]. With the number of people living longer increasing, a new set of challenges arises that needs to be overcome to support the population into old age. The natural decline in physical and mental capabilities as we age poses a serious threat to the quality of life of older adults—particularly when society is not currently equipped to effectively cater to these declines in a way that supports healthy aging [2].

**Figure 1:**
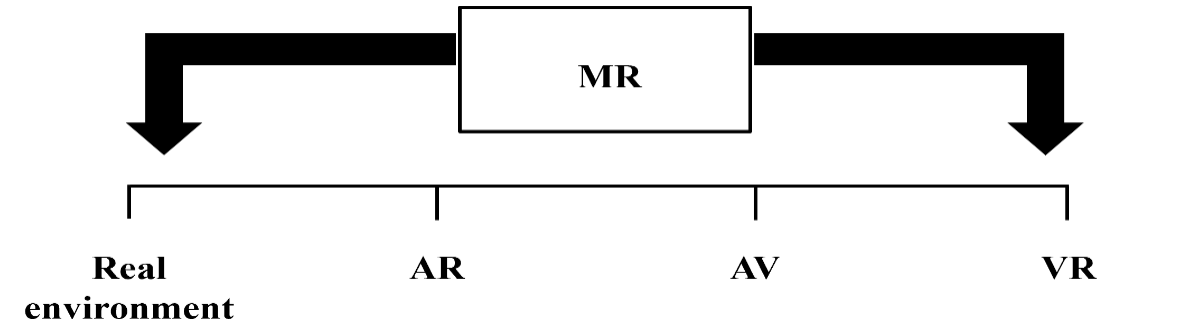
Reality virtuality continuum (adapted from Milgram et al [4] with permission from the authors). AR: augmented reality; AV: augmented virtuality; MR: mixed reality; VR: virtual reality.

The current digital age offers new opportunities to support healthy aging in older populations. A digital technology that has evolved rapidly in the past 10 years is immersive virtual reality (IVR) [3]. On the basis of the reality-virtuality continuum by Milgram et al [4] (Figure 1), IVR is defined as fully computer-generated environments that are shown on a head-mounted display (HMD). IVR sits on the virtuality end of the reality-virtuality continuum. The reality end of the continuum refers to the real environment in which no computer-generated content is overlaid. Between these 2 ends are augmented reality–based displays, where digital information is overlaid onto the real environment through devices such as see-through HMDs, mobile phones, and computer monitors. Slater and Sanchez-Vives [5] described the technical goal of IVR as its ability to “replace real sense perceptions by the computer-generated ones,” simulating what is known as presence and immersion. Presence refers to the feeling of being present in a place (ie, in a virtual environment), and immersion refers to the level of intensity with which one feels that they are present in that place afforded to them by the technological capabilities of the IVR device [5]. Closely linked to presence and immersion are colocation and copresence, which refer to networked virtual environments that enable IVR participants to interact with others (colocation) and feel as if they are there with them in the virtual environment (copresence) [6]. IVR participants are represented in IVR by avatars, which can be described as “human-like machines” that represent the participant in the virtual environment [7]. This representation is known as embodiment, which refers “...to the process of replacing a person’s body by a virtual one” [5].

In their scoping review, Hughes et al [8] discussed how features of IVR such as presence and immersion can assist older adults in their everyday lives, providing opportunities for health promotion and tackling social isolation and loneliness. IVR offers older adults the opportunity to take part in physical activities from their own home that would otherwise need to be facilitated outdoors (eg, virtual cycling in nature [9]). The comfort and convenience of activities such as these being facilitated in a more accessible environment can offer greater motivation for older adults to adhere to health-promoting activities such as physical exercise [8]. Through the incorporation of avatars into networked virtual environments, IVR also offers older adults the opportunity to connect with others in a more meaningful way compared with other communication mediums [6] where meeting in person may not be possible—a reality forced upon many of us during the COVID-19 pandemic [7]. These features align closely with the goals of the WHO for healthy aging [1], particularly with regard to age-friendly practice [1,10]. Age-friendly practice emphasizes the importance of supporting older adults in maintaining a fulfilling and meaningful life into old age through supportive infrastructures in the environment and society. IVR provides an entirely new set of virtual infrastructures that support an environment in which older adults can connect with family, friends, and other members of society [5].

With the emergence of new, high-quality IVR technologies that are now commercially available in higher-income countries, researchers have begun to examine older adults’ experiences and perceptions of IVR. A number of recently published systematic reviews and systematic review protocols have aimed to synthesize the quantitative literature on this topic [11-13], focusing primarily on IVR’s effectiveness, efficacy, and feasibility in various clinical populations. In recent years, we have also seen an increase in qualitative studies examining older adults’ experiences and perceptions of IVR [14-16]. However, to our knowledge, there has been no systematic synthesis of these studies to inform the design of new, more accessible IVR devices for older adults.

In digital technology development and design, qualitative feedback from end users can be invaluable. It provides developers and other informants with rich information to work with when designing digital technology content, with particular utility in identifying various barriers to and facilitators of using a technology [17]. It also offers the opportunity to explore more deeply whether the end user finds a technology acceptable—which is now considered a key factor in determining whether a technology will be adopted and used by the intended user [18]. When defining technology acceptance, it is important to acknowledge the temporal nature of the term [18], with acceptability defined as one’s perception of a technology before use [19], acceptance defined as one’s perception of the technology after initial use [19], and adoption defined as a multiphase process starting with “deciding to adopt (selecting, purchasing or committing to use it) and then achieving persistent use” [20].

Using the Sample, Phenomenon of Interest, Study Design, Evaluation, and Research Type tool (Textbox 1) [21], the following research questions were formulated to guide the review and synthesis of the existing literature: (1) What are older adults’ experiences and perceptions of IVR? (2) What are the barriers to and facilitators of older adults’ use of IVR? (3) Do older adults find IVR acceptable?

**Sample, Phenomenon of Interest, Study Design, Evaluation, and Research Type (SPIDER) tool for defining research questions and search terms.**

**SPIDER constructs and description**

- Sample: in this review, the sample of interest was older adults aged ≥60 years.
- Phenomenon of interest: the phenomenon of interest in this case was older adults’ experiences and perceptions of immersive virtual reality (IVR). The experience of presence, described by Slater and Sanchez-Vives [5] as the feeling of being present in the virtual world with the belief that the events occurring there are really happening, is a key characteristic of IVR that enables the enhancement of the virtual experience. As one removes the sensory substitutions that enable this sense of presence, such as a head-mounted display and haptic devices, the experience changes drastically for the user, making it more challenging to link the qualitative experience. Therefore, as each of the technologies across the reality-virtuality spectrum provide different experiences for the user, it was decided that adhering to the definition of IVR by Milgram et al [4] would provide a more meaningful and translatable qualitative synthesis.
- Design: the study designs searched for in this review used qualitative research methods such as focus groups and semistructured interviews.
- Evaluation: as this review was interested in individuals’ experiences of interacting with an object, terms such as “acceptability” and “usability” were included to identify studies.
- Research type: qualitative and mixed methods studies were searched for.

### Objectives

The objective of this study was to systematically review and synthesize qualitative studies exploring older adults’ experiences and perceptions of IVR.

## Methods

A protocol detailing the background, rationale, and methods of this systematic review has already been published [22]. This systematic review and thematic synthesis were completed following the ENTREQ (Enhancing Transparency in Reporting the Synthesis of Qualitative Research) guidelines [23].

### Search Strategy

A detailed search strategy was developed to identify studies relevant to the review questions (Multimedia Appendix 1). After consulting with a librarian at the university and examining databases used in previous reviews with similar research questions to this review [11,13,24-30], 3 databases were selected to run the search in: Embase, Compendex, and Scopus. These databases were selected as they covered key fields relevant to this review, including computer science, engineering, human-computer interaction, psychology, and health and social sciences. The search strategy was developed for Embase and adapted where necessary for the other databases. The search terms were informed by previous systematic review search strategies with similar research questions [13,24-30] as well as input from the review team. Relevant keywords and phrases were used in each database, including *older adults*, *virtual reality*, *perceptions*, and *experiences*. The search terms were organized into relevant categories using the Sample, Phenomenon of Interest, Study Design, Evaluation, and Research Type tool [21] and then combined into a single search strategy. In some databases, certain categories were omitted from the final search strategy to broaden the scope of studies captured by the search. To ensure that the replicability of these searches is possible, these omissions can be examined in the link provided in Multimedia Appendix 1. Owing to the dramatic evolution of IVR equipment in recent years [3], databases were searched for relevant studies published in English from January 2012 to July 2020—an approach also taken in a recently published systematic review exploring IVR [13].

### Inclusion and Exclusion Criteria

Studies were included if they examined older adults’ experiences and perceptions of IVR. As there is no generally accepted definition of older adults, we included those studies in which the mean age of the study sample was ≥60 years as this is a commonly used cutoff in aging research [31]. Only studies in which complete visual immersion was facilitated through the use of an HMD were included. Studies were included if a qualitative method was used for both data collection and analysis, they were peer-reviewed publications, and they were written in English.

Older adults with a diagnosed neurodegenerative disorder were excluded as there is evidence that their experiences with virtual reality—as well as the application of virtual reality in this cohort—differ considerably from neurotypical individuals’ experiences [32]. Reviews, conference abstracts, opinion pieces, gray literature, and editorials were excluded.

### Screening and Data Extraction

The initial search was conducted by one reviewer (DH). The screening phase of this review consisted of title and abstract screening, full-text screening, and forward and backward citation searching of the included full-text articles. Title, abstract, and full-text screening was completed for studies identified through forward and backward citation searching.

Titles and abstracts were extracted from the chosen databases and combined in EndNote X9 (Clarivate). Duplicates were removed using the Remove Duplicates function in EndNote X9. Records were manually screened for remaining duplicates in EndNote X9. Title and abstract screening was conducted by one reviewer (DH) using the Rayyan (Rayyan Systems, Inc) data screening tool [33]. A random sample of 20% (491/2455) was also screened by a second reviewer (AF). A Cohen κ statistic of 0.96 was calculated, indicating almost perfect agreement between reviewers [34]. Where disagreements arose, unresolved cases were discussed with a third reviewer (JW, OC, or JMS depending on the expertise required) until an agreement was reached. Full-text screening was completed by 2 independent reviewers (DH and AF). Where disagreements arose, unresolved cases were discussed with a third reviewer (JW, OC, or JMS depending on the expertise required). Forward and backward citation searching was completed for all the studies included after full-text screening. This search was conducted by DH. Title, abstract, and full-text screening of the studies identified through forward and backward citation searching was completed by 2 reviewers (DH and AF).

Data extraction was completed following a data extraction protocol developed by DH (Multimedia Appendix 2), with feedback from the rest of the review team. In total, 2 reviewers performed the data extraction (DH and AF) using a prespecified data extraction checklist [22]. The extracted data were organized and analyzed using the NVivo software (QSR International) [35].

### Quality Assessment

The Critical Appraisal Skills Programme tool [36] for qualitative research was used to appraise the quality of individual studies. No studies were excluded based on this appraisal. However, the outcomes of the appraisal were noted for each study and used to inform the synthesis of findings. The Grading of Recommendations Assessment, Development, and Evaluation-Confidence in the Evidence from Reviews of Qualitative Research (GRADE-CERQual) approach [37] was used to assess the confidence that can be attributed to the evidence informing each individual review finding, with ratings of high, moderate, or low confidence being attributed to each finding.

### Thematic Synthesis

A thematic synthesis [38] was conducted to synthesize the data extracted from the included studies. Thematic synthesis is derived from the qualitative method, thematic analysis [39-41]. Several interpretations of thematic analysis are cited by authors when discussing how they conceptualized thematic synthesis. Thomas and Harden [38] state that their approach “concurs with [Boyatzis’s] conceptualization of thematic analysis”—where thematic analysis is not defined as a qualitative method in its own right but as “...a process that can be used with most, if not all, qualitative methods...” [41]. This concurrence is due to the fact that their approach to thematic synthesis incorporates multiple other established methods as well as techniques commonly described as thematic analysis [38].

For this synthesis, the interpretation of thematic analysis (or reflexive thematic analysis [42]) by Braun and Clarke [39] informed the synthesis approach, structured within the stages of the thematic synthesis by Thomas and Harden [38]: (1) line-by-line coding of the extracted data from each included study, (2) grouping of these codes to form descriptive themes that remain close to the data presented in each included study, and (3) going beyond these data to create new interpretations or theories (analytical themes) of the combined studies. These themes were then formed into a coherent narrative and reported in the Results section. A table detailing exactly how the steps by Braun and Clarke [39] were mapped onto the stages of thematic synthesis can be found in Multimedia Appendix 3.

### Reflexivity

Qualitative research is generally considered a subjective process [43], meaning that it is essential to be reflexive throughout it. Authors must reflect on how their perspectives, experiences, and worldviews influence the qualitative process. In total, 3 authors have backgrounds in health psychology (DH, JW, and JMS), two of whom are experts in their fields (JW and JMS); one author is a qualified occupational therapist and human-computer interaction researcher (AF); and one author is an expert in computer science (OC). During the review process, the authors’ preconceptions about the topics being discussed were considered when making key decisions relating to the review, analysis, and write-up. The lead author kept a reflexive journal of the review, analysis, and write-up processes as a record of the critical evaluation of the authors’ influence on the study.

### Protocol Deviations

Owing to the amount and depth of data analyzed in this review, the analysis predominantly focused on only one of the primary review questions: *what are older adults’ experiences and perceptions of immersive virtual reality?* The 2 other review questions were formed into secondary review questions—*what are the barriers and facilitators to older adults’ use of immersive virtual reality?* and *do older adults find immersive virtual reality acceptable?*—and were addressed to a lesser extent. This deviation is in line with the assertion by Braun and Clarke [43] that qualitative research questions can evolve as the research study progresses as a greater understanding of the data being analyzed is formed.

## Results

### Search Results

**Figure 2:**
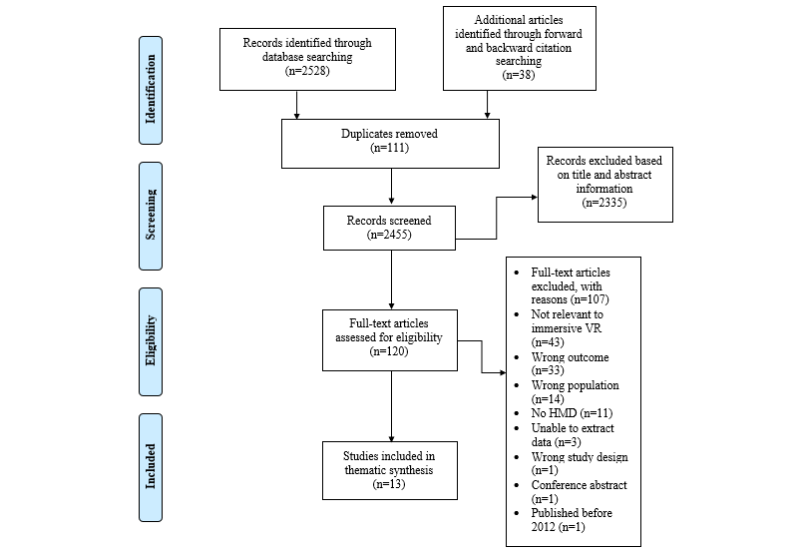
PRISMA (Preferred Reporting Items for Systematic Reviews and Meta-Analyses) flow diagram. HMD: head-mounted display; VR: virtual reality.

In total, 2528 records were identified through database searching. An additional 38 records were identified through forward and backward citation searching. Of the total 2566 records, 111 (4.33%) duplicates were removed, leaving 2455 (95.67%) records to be screened. A total of 95.11% (2335/2455) of records were excluded based on title and abstract information, leaving 120 articles to be assessed during full-text screening. Upon completion of the screening process, 13 studies were included in the final synthesis. This process has been illustrated as a PRISMA (Preferred Reporting Items for Systematic Reviews and Meta-Analyses) flow diagram in Figure 2.

### Study Characteristics

A total of 13 studies were included [9,14-16,44-52]. In total, 77% (10/13) of the studies explicitly reported conducting some form of qualitative data collection procedure [15,16,44-46,48-52], which included focus groups [44,50], exploratory workshops [14], video recordings [9], and self-reported qualitative data [47]. A total of 224 participants were included across the 13 studies. In the 77% (10/13) of studies [9,14-16,44,46-50] that reported participants’ gender, 73 (37.62%) participants were men and 121 (62.37%) were women. The participant age range across the 13 studies was 48 to 99 years. Participants lived across 9 different countries (Australia [14,15,45], China [16,47], United States [44,50], Denmark [9], Northern Ireland [46], Brazil [48], England [49], Taiwan [52], and Thailand [51]) and 5 different continents (Australia [14,15,45], Asia [16,47,51,52], North America [44,50], South America [48], and Europe [9,46,49]).

Of the 10 studies that reported the participants’ place of residence, participants from 5 (50%) studies identified as community-dwelling adults [14,16,44,47,50], and participants from 5 (50%) studies reported living in some form of residential aged-care facility [9,15,45,46,50]. Although it could be assumed that almost all the participants were retired or not working as many lived in residential aged-care facilities, only 31% (4/13) of the studies explicitly reported their participants being retired [9,15,45,50]. In total, 85% (11/13) of the studies reported their recruitment strategy, which included recruiting through community organizations and day centers [16,45,46,49,50,52], posting flyers in public places [14,44], and recruiting through a residential aged-care facility [15] and a physical therapist [9], as well as 8% (1/13) of the studies that described recruiting participants sequentially [48]. Of the 5 studies that reported participants’ mental status, 1 (20%) provided a detailed report on 2 of its participants, with one participant described as being depressed, self-isolating, having a history of behavioral issues, and having mild cognitive impairment and the other participant described as also having mild cognitive impairment [15]. Participants from 23% (3/13) of the studies were generally described as cognitively healthy [9,46,48]. It was reported in 8% (1/13) of the studies that participants were not screened for cognitive impairment, but 3 participants disclosed during focus groups that they had “some dementia” [50]. In total, 46% (6/13) of the studies reported the health status of their participants, with 17% (1/6) reporting some participants being wheelchair users [15], 17% (1/6) having some participants who used walking aids and had a high risk of falling [46], 50% (3/6) reporting participants as having fair health or sufficient health to take part in the activity [9,44,48], and 17% (1/6) reporting cases of arthritis [45].

A total of 54% (7/13) of the studies reported the location where the study took place, including a day center [46,49]; a residential aged-care facility [15]; a community activity center [16]; a laboratory setting [44]; a physical therapy clinic [9]; and a room that had a flat, even floor surface with a trackable area of 2.4×2.4 m [45]. In total, 69% (9/13) of the studies reported participants’ previous technology experience or proficiency [9,14,15,44,45,48-50,52], with experience and proficiency across the studies ranging from high to low.

There was also a range of activities in which participants from each study took part during their IVR experience. The characteristics of these activities are included in this section to further contextualize the experiences referred to in the descriptive and analytical themes reported in the following sections. The specific applications through which the participants completed these activities can be found in Multimedia Appendix 4 [9,14-16,44-54]. The types of activities older adults engaged in across these studies included travel and exploration [15,16,44,45,47,50,51], social connection [14,15,44,48,50,52], entertainment [15,47,49,50], exercise [9,46,48], education [14,47,50], and reminiscence [44,48-50]. Descriptions of these activities and supporting author and participant quotes can be found in Multimedia Appendix 5 [9,14-16,44-52].

### Quality Appraisal

**Figure 3:**
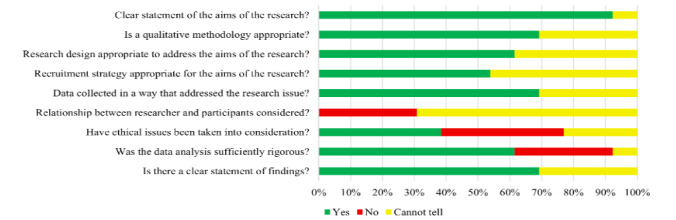
Methodological quality assessment of included studies.

A summary of the methodological quality assessment of the included studies using the Critical Appraisal Skills Programme tool is shown in Figure 3, with full details in Multimedia Appendix 6 [9,14-16,38,39,44-52]. All but 8% (1/13) of the studies [49] had a clear statement of their aims. It was unclear whether the qualitative methodology was appropriate in 31% (4/13) of the studies [9,47,51,52] and whether the research design was appropriate in 38% (5/13) of the studies [9,47,49,51,52]. It was unclear whether the recruitment strategy was appropriate in 46% (6/13) of the studies [45,47-49,51,52] and whether the data were collected in a way that addressed the research issue in 31% (4/13) of the studies [45,47,51,52]. The main reason for this uncertainty was that the authors did not clearly justify the rationale for their methodology, research design, recruitment strategy, or data collection technique.

In 31% (4/13) of the studies [47,49,51,52], there was no consideration of the researchers’ relationship with the participant, and it was uncertain in the remaining studies [9,14-16,44-46,48,50]. No ethical concerns were considered in 38% (5/13) of the studies [9,45,47,51,52], and data analysis was considered not sufficiently rigorous in 31% (4/13) of the studies [46,47,51,52].

### Confidence in the Evidence

Confidence in the evidence was assessed using the GRADE-CERQual [37] tool and ranged from high to moderate (Multimedia Appendix 7 [9,14-16,44-52]). There was consistency in the findings across countries, continents, and settings. Ratings of moderate certainty were mostly due to methodological limitations and adequacy. The most common methodological limitation was the lack of reflexivity regarding the relationship between the researchers and participants. The main concern regarding adequacy was the lack of rich data informing a number of the findings.

### Overview of Thematic Synthesis

Three descriptive themes were generated that were related to older adults’ experiences and perceptions of IVR: (1) practical aspects of IVR use, (2) experiencing unique features of IVR, and (3) perceptions of IVR. Textboxes 2 to 4 include illustrative quotes for each descriptive theme and its respective subthemes. Author quotes are italicized to distinguish them from quotes provided by research participants in each of the studies.

Three analytical themes were then formulated by interpreting key patterns present across the descriptive themes, generating new meaning from the synthesis: (1) tolerating the bad to experience the good, (2) buying into IVR (don’t judge a book by its cover), and (3) “it proves to me I can do it.”

**Illustrative quotes for the descriptive theme of practical aspects of immersive virtual reality (IVR) use and its subthemes.**

**Interacting with IVR hardware and software**

- “I would never wear anything that heavy to watch something.” [50]
- “But I think this device is worn on the head, and there is something on the head that always makes me feel uncomfortable.” [16]
- “with the headset the movement was throwing me off a bit I think, and making me feel sort of disorientated and dizzy.” [46]
- “...[the HMD is] in need of improvement in ways such as accommodating larger glasses...” [50]
- “*Some indicated that the smoothness of the plastic made it difficult to press track and menu buttons with confidence.*” [45]
- “*Participants stated that whilst at first they could remember buttons they forgot during the main exercise and became frustrated because there were too many option [buttons] to choose from.*” [45]
- “*They [participants] did not report having many problems and even enjoyed this aspect [handheld controllers], as it added a sense of control.*” [44]
- “*Some [participants] found that background noise and conversations from other people in the room was distracting and disconcerting.*” [45]
- “*Many felt VR is promising, but in need of improvement in ways such as* ‘...increasing the crispness of images.’” [50]
- “In terms of image, in terms of quality, in terms of sound, in terms of perception, virtual reality is much better.” [48]
- “There is so much to watch here!...It’s amazing to look at the birds.” [9]
- “She [virtual avatar] asked me if I wanted to dance...well...she’s...invited me to dance and asked me to be careful with the chair.” [48]

**Risks and requirements of IVR**

- “I would prefer to have somebody [...] Well for instance you saying to me ‘Sit down, the chair is right behind you.’ You know it is going to be there, but still, it gives you the confidence to know that somebody is actually telling you that.” [46]
- “*Because of physical problems, such as weak vision, high blood pressure, and motion sickness, some elderly people reported to feel dizzy and other discomfort. At the same time, the equipment is still relatively cumbersome, and it may be uncomfortable to wear on the head.*” [16]
- “I can’t see well. This is a place to pay the homage [sic] but I can’t see the prayers. Can I wear glasses?” [51]
- “It’s difficult for older people to turn...you really have to turn your whole self.” [50]
- “Waving swords is too intense for me. Maybe something gentler, you know, like picking apples.” [47]
- “As you get older, you’re less mobile and you can see...you can bring people together in [social VR]...you’d [feel] ‘I’m not going to get on the bus...but let’s get together in [social VR].’” [14]
- “Because of my age, I can’t visit some attractive places. [Even though] I want to, I don't dare to go and my children will be worried about it. [But] If you watch it [VR], it is a kind of enjoyment as well as filling in an inner gap for yourself.” [16]
- “if I don’t do something consistently, I have to go back and write it down and have the directions written.” [50]
- “I found the system, once it was explained to me, it was quite simple to use. It was quite easy.” [46]

**Illustrative quotes for the descriptive theme of experiencing unique features of immersive virtual reality (IVR) and its subthemes.**

**Presence and immersion**

- “*At the conclusion of his first session using the underwater discovery game ‘Ocean Rift,’ Neville commented that* ‘it really feels like you are underwater.’” [15]
- “There was simply...more...You completely get the sensation what you were actually driving inside the landscape...You are out in the middle of it all! Can you believe it?” [9]
- “I also like it, the feeling of being there.” [16]
- “Visualization is better, the participation, you’re more inside than on the cell phone, as if I were in the living room of my house.” [48]
- “[virtual reality] removes you from your thoughts and your worries and, and you don’t hear the phone ring and you...don’t have any distractions at all...” [50]
- “It was just like being on a roller coaster. It was really good. Every bit, every bump you can actually feel.” [49]
- “It’s supposed to be a circus, I guess, but...I didn’t feel it was a circus.” [50]
- “to get any real benefit out of [social VR], you’d have to believe that [the avatar represented] that person. They would have to show some sort of emotion...there wasn’t the interaction.” [14]
- “Wow, 100%, music from my time, it all helped!...I saw my daughter there dancing on the rug, as if I were in my living room.” [48]
- “Then when I went, I went into the dance, I started shaking my head so I do not know if...I felt a certain discomfort...maybe even a little bit of nausea.” [48]

**Sensations and emotions experienced in IVR**

- “With my ears listening and my eyes watching, I forget about the unpleasant things in my heart.” [16]
- “It’s amazing to look at the birds...Oooh, now I’m getting dizzy [from looking over the edge]...It feels like I’m really about to go downhill!” [9]

**Embodied experiences in IVR**

- “I mean you could do [any movement] with your fingers and it didn’t show up [on your avatar].” [14]
- “...to get any real benefit out of [social VR], you’d have to believe that [the avatar represented] that person. They would have to show some sort of emotion...there wasn’t the interaction.” [14]
- “*While VR is often described as an individual experience, our results suggest that VR can act as a powerful tool to provoke social interaction and thus counteract the high levels of social isolation*...” [15]

**Illustrative quotes for the descriptive theme of perceptions of immersive virtual reality (IVR) and its subthemes.**

**Preconceptions of IVR**

- “I really didn’t think it would be anything that I would enjoy, and I certainly didn’t think it was something I could use, however I was intrigued to find out about virtual reality was like.” [45]

**Perceptions of IVR during and after use**

- “This technology is very good, I think it encourages more...it is a thing with a good look, I would give 10 for the glasses, I found it very good, great!” [48]
- “This is what I’ve been wanting all along.” [9]
- “What I did here for me today was new. I never did it.” [48]
- “I liked the glasses better because it is different, right?” [48]

### Descriptive Themes

#### Practical Aspects of IVR Use

This theme was discussed in 13/13 of the studies and illustrates older adults’ experiences interacting with IVR hardware and software as well as the challenges that arise when facilitating IVR interactions for them.

##### Interacting With IVR Hardware and Software

Participants in 11/13 of the studies reported issues with the HMD, including that it was too heavy [9,44,45,48,50]; caused general discomfort [9,15,16,44,50]; caused disorientation and imbalance [44,46]; and caused feelings of being trapped, confined [45], afraid, or anxious [16,45]. Difficulties with the HMD being too small to wear with glasses were also described [14,44,46,48,50,51]. Although a range of issues were raised regarding the HMD, 3/13 of the studies reported that the HMD was generally tolerated by participants [9,14,15].

Issues with the handheld controllers were reported in 5/13 of the studies and included poor ergonomics of the controllers [15,45] and the awkward feeling and positioning [15,45] and smoothness of the buttons [45]. The number of buttons also caused problems, with participants forgetting the function of each of them over time [45]. Issues with handheld controllers were not described in all studies, and some studies reported that participants could use the handheld controllers confidently [15,44] and enjoyed the added control that the handheld controllers gave them in the virtual environment [44]. Of note, the number of buttons that participants had to use was not reported in the extracted data, making it difficult to compare across studies.

Perceptions of the audiovisual quality of IVR hardware varied across studies [14,16,45,48-52]. A total of 5/13 of the studies reported some form of audiovisual issue [14,45,49-51], with feedback that the volume was not at the right level or was not clear enough [45,49-51] or that sound coming from the physical environment could negatively affect the IVR experience [45]. Older adults believed that the resolution of the display must be of high enough quality to ensure a positive experience [50,51]. A study raised the issue that only the participant could be sure if the lenses were fitted correctly, with the researcher never being certain whether the image they were viewing was unclear or just not fitted correctly on the participant’s head [14].

In total, 6/13 of the studies reported feedback from participants on how the body tracking in IVR influenced their experience [14,15,44,48,50,51]. Issues included feelings of nausea and dizziness [48] and the virtual handheld controllers disappearing from the screen in the virtual environment [44]. Ensuring that the geopositional tracking does not negatively affect the participant was also discussed, with some participants feeling that the origin of the sound was confusing and that moving content made it more difficult to hear audio while also increasing feelings of nausea [14,51].

A total of 11/13 of the studies described feedback on the content presented, specifically the various objects and scenes inside the virtual environment and participants’ experiences navigating them [9,14-16,44,46-48,50-52]. Participants preferred still content to moving content [44,51] and preferred content that was familiar [52], in particular, content similar to past experiences in real life [44]. An important suggestion made was customizing the moving content experienced in the virtual environment to offset the effects of motion sickness [51]. Participants showed a strong preference for tailoring the content to their own interests [15,16,47]. They enjoyed the adaptive nature of the content [15], suggesting also that there could be more “diversity” in the content displayed in IVR, although it was not specified what exactly this content could be [52]. There were only a few negative comments about the content, with some content causing nausea or being too intrusive or incompatible with preferences [16,50,51].

In 4/13 of the studies that described participants’ experiences navigating the virtual environments, participants had little issue in doing so [44,48,50,52]. Participants found it easier to interact with the virtual environment after a few practice attempts [52] and also once the equipment was fitted correctly as this enabled them to focus on using the handheld controllers [50]. Participants were also able to interact with the virtual objects such as other virtual avatars [48,50], with a participant explaining that a virtual avatar was inviting her to dance with them [48].

##### Risks and Requirements of IVR Use

With regard to health and safety [9,14-16,44-46], it was clear that older adults found it dangerous to use an HMD on their own as they were unaware of their physical surroundings when wearing it [9,46] and would like to have someone present to reassure them that they were safe [46]. Self-awareness of HMDs that were tethered to a computer also caused concerns as participants were worried that they would damage the cable or be electrocuted if they stood on it [45]. Weak vision, high blood pressure, and motion sickness brought about feelings of dizziness and discomfort for some [16], whereas others felt unsteady when using the equipment [44]. However, in another study, participants reported not feeling any dizziness or nausea during their IVR experience [14].

Specific risks for wheelchair users described in 1/13 of the studies included overreaching for an object in the virtual environment and falling from their chair, injuring their arms when interacting with the virtual environment because of high armrests, and releasing the brakes of the wheelchair to allow for more movement causing simulator sickness [15]. The authors recommended ensuring that the chair or wheelchair was positioned correctly so that participants could reach virtual objects without having to overreach and risk falling [15]. Despite these issues, other studies found that participants reported having a positive experience with IVR from a sitting position [44,50].

The physical capabilities of the participants and how that influenced their experience with IVR were also reported [9,15,16,44,47,48,50,51]. Having impaired hearing or vision hindered the participants’ experience with IVR [16,50,51]. A lack of mobility also prevented some participants from making the most of the full 360° experience [9,47,48,50], where some found it difficult to turn themselves to see other features in the environment [50]. This also highlighted that the experiences need to be tailored to the participants’ own capabilities so that they can experience the virtual environment to its full potential [47,48], particularly when people have varying levels of capabilities regarding their use of IVR equipment [15]. In one case, being able to visit places where they could not physically go themselves brought about a heightened sense of immobility [16]. However, in contrast, participants also reported that IVR could give back agency lost through aging because of physical and mental decline [14,15,50], affording them the opportunity to explore places and take part in activities that, if they were to do in reality, might raise concerns about safety among their family members and other key stakeholders [16].

The need for assistance when using IVR was also apparent [44,46,50]. Participants were concerned that they would not remember how to use the technology at a later stage [50]. The authors voiced some concern that participants would need assistance in setting up the equipment at this later stage [50]. Where reported, participants with greater previous digital technology experience needed less support using the equipment than those with less experience [44]. However, even participants with less experience found the technology easy to use once it was explained to them [46].

#### Experiencing Unique Features of IVR

This descriptive theme consists of 3 subthemes exploring participants’ experience of presence and immersion, the emotions experienced in IVR, and their experience of embodiment.

##### Presence and Immersion

A total of 10/13 of the studies reported participants’ experiences and perceptions of presence and immersion in IVR [9,14-16,46,48-52]. Participants reported that the feeling of presence made the experience feel more real [15] and allowed them to engage with objects in the virtual environment as if they were really present [9]. Participants from 3/13 of the studies explicitly reported enjoying the experience of presence [9,16,48]. By contrast, a number of participants in 1/13 of the studies had some feelings of anxiety and nervousness because of the experience of presence in the immersive environment, although no specific reason for these feelings was given [16].

A total of 1/13 of the studies reported that the more participants were immersed in the virtual environment, the more it enhanced their experience [48]. Greater immersion provided a heightened travel experience when exploring places around the world [15,16], enabled participants to escape from their own reality [50], and simulated past experiences in a realistic way [49]. Some participants were not convinced by the immersive nature of the technology and felt that they were “passive observers” as they did not feel as if they were in the place they were supposed to be [50]. Participants needed to be able to interact with the content in the virtual environment to make the experience realistic and believable [14], and more familiar virtual environments and situations made the experience more real [48]. Many participants reported really enjoying the immersive nature of IVR [9,14,16,48,52], with only one participant reporting negative sensations because of the immersive experience [48]. This participant also reported enjoying their experience with IVR, suggesting that the positive effects of the experience offset the negative sensations felt.

##### Emotions Experienced in IVR

Emotions experienced by participants were referred to in all studies (13/13) [9,14-16,44-52] and appeared to be a central part of the IVR experience.

Participants from 8/13 of the studies reported experiencing some form of positive emotion because of their interactions with IVR [9,15,16,44,45,48,49,51]. Some made explicit reference to the fact that it was the immersive experience that incited these positive emotions [9,16,48]. General reports of positive emotional experiences with IVR included it having the “wow” factor [45], having fun interacting with virtual avatars in the virtual environment [15], and always laughing when experiencing the virtual content [51].

Excitement was also common [9,16,45,48,50,51]. In most cases, this excitement referred to a heightened sense of emotion toward the virtual content [9]. In one case, the excitement came to a point where a participant became too giddy and needed to sit down and rest for a period [45].

Negative emotions were also experienced by participants, such as feelings of intrusiveness as the virtual experience felt like an intrusion of their personal space and boundaries [50] and feelings of stress and frustration caused by disassociation with the virtual environment and distrust of the electrical equipment [45]. Regarding this final point, a term was created to describe this combination of experiences, referred to by the authors as “3D fears” [45].

##### Embodied Experiences in IVR

Embodied experiences in IVR were discussed in 4/13 of the studies [14,15,44,49]. Embodiment refers “...to the process of replacing a person’s body by a virtual one” [5]. These embodied experiences relate specifically to how participants interacted with their avatar (the body replacing their own body in IVR) and other participants’ avatars in a virtual environment as well as how being embodied in an avatar in a virtual environment made them feel.

Where given the opportunity, participants enjoyed creating their own avatars and embodying them in IVR [14]. However, for this experience to be enjoyed, a number of key issues must be addressed. Glitches in body tracking led to concerns over the negative stereotypes associated with aging. A participant felt as if he had developed Parkinson disease when his virtual hands began to shake involuntarily [14]. The authors concluded that their findings illustrated how tracking errors can negatively affect participants’ experiences with IVR when they are trying to express social meaning through nonverbal cues. A critical point closely related to this was that tracking errors also led to participants feeling as if they did not have control over their avatars and, by extension, their own bodies, making them “...particularly sensitive to social stereotypes that render the ageing body as being an object of disgust that makes them ‘liable to sanctions, both physical and symbolic’” [14]. Finally, participants thought that the avatars were not realistic enough to facilitate a meaningful social interaction [14].

The power of embodiment and its implications for socialization were also linked to alleviating social isolation [14,15]. However, the authors 1/13 of the studies highlighted that, although embodiment in a virtual environment for one individual may alleviate social isolation and loneliness, it may also emphasize the limitations that another individual might have if they are not capable of exploring the scenes presented in the virtual environment in reality [44].

#### Perceptions of IVR

This descriptive theme explores older adults’ perceptions of IVR, with the first subtheme outlining perceptions before use and the second outlining perceptions during and after use.

##### Preconceptions of IVR

Most of the preconceptions reported were negative [15,44,45], with a general sense that IVR was a “frivolous undertaking” with little benefit to older adults and better suited to younger generations [45]. Some participants worried that they would forget how to use the handheld controllers [15], whereas others were concerned about whether their glasses could be worn with the equipment and, if so, whether the HMD would damage the lenses of their glasses [44]. In contrast, participants from 1/13 of the studies hoped that IVR would “broaden their horizon” [47].

##### Perceptions of IVR During and After Use

This subtheme reports specifically on participants’ overall perceptions of the technology rather than on any specific feature [9,14-16,44-46,48-52].

Feedback provided by participants during and after use was mostly positive [9,14,16,46,48,49,51]. Authors from 1/13 of the studies reported that participants were excited and curious about IVR and were keen to learn about its potential benefits [14]. A participant from another study went as far as to say that it was a technology that had been missing from their life [9].

Novelty experienced in IVR was identified as a distinct pattern in the data [16,48,50,52], usually referring to the unique features IVR has that other technologies do not, such as the ability to completely immerse a participant in a virtual environment [16]. In general, although not explicitly stated in some cases, this novelty referred to positive experiences that participants had with IVR [48].

A number of negative responses to IVR were also reported [16,45,50]. Some participants preferred technologies that did not require an HMD [50]. Others said that they already had enough devices in their lives and did not need another one [50]. The authors of 1/13 of the studies reported that participants found IVR too overwhelming [45], which was supported by additional feedback suggesting that IVR was not suitable for older adults [16].

### Analytical Themes

A total of 3 analytical themes were developed based on the descriptive themes to go beyond what was reported in the original studies to generate new meaning and tell a story about older adults’ experiences and perceptions of IVR.

#### Tolerating the Bad to Experience the Good

A focus of the descriptive themes related to older adults’ interactions with IVR as well as how these experiences were facilitated (practical aspects of IVR use [9,14-16,44-52]). These accounts included a range of issues that arose while interacting with both the hardware and software as well as positive and negative accounts of the IVR experience (experiencing unique features of IVR [9,14-16,44-52]). On the one hand, many of the negative accounts could be perceived as disincentives for future IVR use that offer little redeeming features for older adults to revisit. However, when considering many of the participants’ experiences as a whole, it was clear that a few changes to facilitate their experience with this technology could greatly enhance their interactions with it. For instance, although some issues relating to comfort and usability were raised, a keyword used in 1/13 of the studies to describe how these issues were experienced was “tolerable” [14]. Many participants across these studies were willing to tolerate some of the discomforts that accompany existing IVR technologies to experience features such as immersive networking with other older adults [15]. A striking pattern in the data was the fact that, despite the numerous issues raised by participants, the novel experiences that IVR afforded them in many cases outweighed the nuisances caused by the equipment.

#### Buying Into IVR: Don’t Judge a Book by Its Cover

There was a temporal pattern identified in the data that consisted of older adults’ perceptions of IVR across stages of IVR use (perceptions of IVR [9,14-16,44-52]). It began with older adults’ perceptions of IVR before use, followed by their perceptions during and immediately after use. This pattern illustrates the importance of users’ preconceptions of a given technology and how they can differ from their actual experience with it. The participants’ perceptions after use provided greater insight into what older adults like and dislike about IVR and how the experience can be improved before future use.

It was clear that participants generally had low expectations for their experience with IVR, with concerns that they would not be able to use it or that it would simply be a pointless endeavor as they believed that they would have no use for it in their everyday lives [45]. However, once participants had experienced IVR, there was a notable change in their views toward it. Many participants were excited about the opportunities IVR offered them in their lives, enjoying the various novel features such as the 360° view and networked activities [15,16]. This change in impression voiced by many of the participants suggests that there is a discrepancy between how IVR is perceived before and after use, with a generally negative impression of the technology before use changing to a generally positive impression after use.

#### “It Proves to Me I Can Do It”

This analytical theme conveys the added agency that IVR afforded older adults in several studies. It draws on the wide range of activities that IVR offered to older adults and how these activities appeared to increase agency in this cohort, which was apparent across almost all the descriptive themes (practical aspects of IVR use [9,14-16,44-52] and experiencing unique features of IVR [9,14-16,44-52]). It illustrates an important point about the need to give back agency lost by older adults with growing frailty and immobility as well as the transition from independent to assisted living: “It proves to me I can do it [participate in an IVR activity], it’s been a long while since I did anything like that” [46]. The activities that older adults in these studies experienced and shared their views on highlight that IVR provides a platform for older adults to access certain activities and environments more easily than in the real world because of limitations caused by aging, as well as providing activities that they are able to follow and take part in as they are tailored to their needs. It was clear that this freedom to take part in these sought-after experiences in IVR on their own terms was an important feature of IVR for many participants. As such, IVR is conveyed as a pathway to this increased agency in this cohort.

## Discussion

### Principal Findings

This review synthesized 13 qualitative studies exploring older adults’ experiences and perceptions of IVR. The thematic synthesis explored older adults’ experiences interacting with IVR and what challenges arise when facilitating their use of IVR; the unique features of IVR that older adults experienced, such as presence, immersion, and embodiment; and older adults’ overall preconceptions of IVR and perceptions of IVR during and after use. The confidence that could be attributed to each finding, assessed using the GRADE-CERQual approach, ranged from high to moderate, with most findings given a moderate rating.

This review did not intend to be exhaustive in its interpretation of all these topics, nor did it intend to offer an exhaustive list of design considerations for future IVR use in this population. It aimed to tell a story about the key findings of this synthesis that other researchers can draw on when designing IVR experiences for older adults. It aimed to go beyond the practical design considerations already offered in papers such as that by Abeele et al [55] to provide a more empathetic interpretation of the experiences older adults have had with IVR to date. By *empathetic*, we refer to the researcher or technology designer putting themselves in the shoes of the end user—in this case, older adults—when exploring these individuals’ experiences and perceptions of a technology [56]. This approach responds to the WHO’s call for more age-friendly practice [1,10], which endeavors to build infrastructures that older adults can avail of that will enhance their quality of life.

### In the Context of Other Research

Conflicting participant experiences were present across the synthesis. These conflicts included differences in older adults’ experiences of dizziness and nausea [14,44], wearing the HMD [9,14,15,44,46,48,50,51], and sitting while using IVR [15,44,50]. These conflicts highlight that there is a need for such features to be adaptive to older adults’ experiences with IVR. This suggests that an assessment of their capabilities could be completed before they use the equipment to tailor the IVR experience to participants’ physical and mental capabilities [47,48]. A key element of this finding is the importance of tailoring the experience to older adults’ needs and preferences. Previous research has generated a list of key design features that can be used to help solve issues identified through older adults’ experiences reported in this review [55].

A striking finding from this synthesis was the level of agency that the IVR equipment afforded older adults while in virtual reality. It was clear that older adults experienced greater levels of agency during their IVR experience, in some cases enabling greater levels of agency than they had in the real world [14,15,44,50]. Parallels between this finding and previous research exploring the applications and implications of IVR can be made, with equipment such as handheld controllers affording participants features such as illusionary agency over the avatar they embody in the virtual environment [5]. This illusory agency affords participants enough autonomy to take part in experiences that may not be available to them in their own reality. Older adults can also be negatively affected by the added agency they experience in IVR, where it may sometimes only emphasize their own limitations in reality [44]. A balance must be struck between what is perceived as a freeing and beneficial activity by older adults through using IVR and what can be perceived as a reminder of their own age-related limitations.

Another salient finding of the synthesis was the change in participants’ generally negative perceptions of IVR before use to generally positive perceptions after use. This is in line with existing research suggesting that the process of accepting a technology resembles a life cycle, with preconceptions of a technology being an essential part of this life cycle as they play a role in whether the participant will eventually adopt the technology [18].

There were clear signs that participants were generally happy to tolerate certain nuisances inherent to some of the existing IVR technology features to experience what they considered to be meaningful activities [15]. Social connection in IVR was one of these sought-after activities for many participants because of the added opportunity it gave them to meet others outside their sometimes restrictive environment [14,15]. These novel interactions experienced by participants incited a newfound sense of excitement, in many cases leading to more motivation to take part in activities in IVR. However, current research supports the need for improvement in existing IVR features to facilitate more meaningful IVR activities such as social interaction [7]. Evidence suggests that older adults appreciate certain features that IVR offers them, such as added anonymity, which encourages introverted participants in particular to share more in IVR social circles [7]. However, IVR is still considered too complicated for older adults to engage with as a means of taking part in these social networking experiences when compared with face-to-face communication as well as other computer-mediated technologies such as FaceTime or Skype [7].

A key finding of this review is the success some researchers had in identifying and offering solutions to some of the nuisances reported by participants through the methodologies they used to explore this topic [7,14,15], such as participatory action research. This approach, along with other co-design approaches such as Patient and Public Involvement [57], can help improve the usability and accessibility of IVR technologies for older adults as the technology continues to rapidly evolve over time. Co-design approaches enable researchers to iterate on versions of IVR hardware and software more rapidly without the need to repeatedly collect and analyze data, which is essential given the rapid turnover of new IVR technologies in today’s market [3].

### Implications and Recommendations

In line with the primary review question—“what are older adults’ experiences and perceptions of IVR?”—this synthesis offers an insight into the experiences older adults have had with IVR and their perceptions of these experiences. The approach to implementing and exploring IVR needs to be taken with care and empathy for the individual [56] as our results demonstrated variation in experiences and perceptions of interacting with IVR. It is hoped that this empathetic perspective offers researchers in this field more direction when considering how to first approach introducing IVR to older adults and later ensuring that their IVR experiences are facilitated in a way that makes it more meaningful for them. This empathetic approach to design is in line with the age-friendly practice of the WHO [1,10], where infrastructures that are put in place to maintain and enhance older adults’ quality of life are designed to specifically support older adults’ needs and preferences.

With regard to the secondary review question “what are the barriers and facilitators to older adults’ use of immersive virtual reality?” the descriptive themes illustrated the barriers older adults face when using IVR, including challenges regarding health and safety when using IVR, wheelchair and stationary use during IVR activities, physical capabilities when using IVR, and the assistance needed to interact with IVR. As there was little reporting on the facilitators of IVR use in the extracted data, future research needs to focus on exploring solutions that can help older adults and other key stakeholders overcome the barriers outlined in the synthesis. With regard to the secondary review question “do older adults find IVR acceptable?” participants’ perceptions indicated that, once they tried IVR, they generally enjoyed the experience despite some of the shortcomings of the technology. Exploring further why exactly participants find IVR acceptable is important in future research as it is now considered a key determinant of whether a technology will be adopted in the future [18].

As outlined in the Introduction section, older adults require greater assistance as they age to continue leading a fulfilling and independent life [10]. The descriptive and analytical themes highlighted that IVR can provide a platform for older adults to access activities and environments more easily than in the real world because of limitations caused by aging, as well as providing activities that they are able to follow and take part in as they are tailored to their needs. Moreover, it was clear that older adults enjoyed and ascribed meaning to their IVR experiences, with reports that, in some cases, the activities in IVR were more appealing than those offered to them in their real environment [15]. Such outlets increased people’s desire to return and try these experiences again as they afforded older adults more opportunities to take part in activities that they were able to engage in and enjoy.

On the basis of the review and synthesis of findings, there are a number of recommendations for future research conducted in this area. First, it is essential that researchers provide a comprehensive report on the nature of participants’ interactions with IVR. There were several cases where it was challenging to compare features across studies as there was not enough detail given on the features that participants interacted with. For example, participants across a number of studies found the handheld controllers difficult to operate, but it was not specified how many buttons participants were using, which is a key consideration when assessing what level of complexity is within certain participants’ capabilities. In line with the outcomes of GRADE-CERQual, authors also need to explore further their relationship with the research participants. Reflexive practice of this nature offers both the authors and readers a greater understanding of the context within which the study was designed and the perspectives of the authors that could potentially influence future interpretations of the findings [43]. The GRADE-CERQual approach also highlighted the lack of clarity regarding ethical considerations taken in several studies. It is essential that researchers report explicitly on these considerations, especially when working with vulnerable cohorts.

### Strengths and Limitations

The review protocol was published [22], registered on PROSPERO, and preregistered on the Open Science Framework, where an open-source repository of all the review materials has been stored and updated (Multimedia Appendix 1). The ENTREQ guidelines were followed when writing the report [23]. The review team had a wide range of expertise, ensuring that the interpretations made in the synthesis were accessible across multiple relevant disciplines. Thematic synthesis was an appropriate method for data synthesis as it allowed the reviewers to stay close to the results of the primary studies, which facilitated “...the explicit production of new concepts and hypotheses” [38]. The search strategy was broad. In total, 2 reviewers screened the studies to reduce bias. This review provides a new perspective on how older adults’ experiences and perceptions of IVR can be interpreted, taking a more empathic and experiential approach to data synthesis rather than focusing on irreducible, quantifiable design considerations.

The exclusion of non-English studies is a potential source of bias. However, because of the lack of available resources, no translator could be used for the non-English studies identified. Gray literature was also not searched, which limited the included studies to published works. The reasoning for excluding gray literature was because a scoping review of the gray literature before conducting the systematic review search indicated that there was limited qualitative data available that were not published in academic journals.

### Conclusions

This review offers an insight into the experiences older adults have had with IVR to date. With a few improvements to existing IVR hardware and software, focusing also on how it is first presented to older adults, IVR may arise as a new outlet through which older adults living both independently and in residential aged-care facility could take part in a range of meaningful activities that are tailored to their needs and preferences.

## Appendix

Multimedia Appendix 1 Embase search strategy and osf link.

Multimedia Appendix 2 Data extraction protocol.

Multimedia Appendix 3 Reflexive thematic analysis mapped onto thematic synthesis.

Multimedia Appendix 4 Virtual reality applications.

Multimedia Appendix 5 Immersive virtual reality activities.

Multimedia Appendix 6 Quality appraisal summary.

Multimedia Appendix 7 Grade-cerqual summary.

## Abbreviations

ENTREQ: Enhancing Transparency in Reporting the Synthesis of Qualitative Research
GRADE-CERQual: Grading of Recommendations Assessment, Development, and Evaluation-Confidence in the Evidence from Reviews of Qualitative Research
HMD: head-mounted display
IVR: immersive virtual reality
PRISMA: Preferred Reporting Items for Systematic Reviews and Meta-Analyses
WHO: World Health Organization
