# Supplementary material for: Older Adults’ Experiences and Perceptions of Immersive Virtual Reality: Systematic Review and Thematic Synthesis
Source: JMIR Serious Games. 2022 Dec 6;10(4):e35802. doi: 10.2196/35802 (PMC9768659; doi:10.2196/35802)
Supplement: Multimedia Appendix 1 [file games_v10i4e35802_app1.docx]

**Search Strategy**

Database: Embase

Search Strategy:

1. 'older adult*'
2. 'young old'
3. senior*
4. elder*
5. aged [preferred term]
6. ag$ing
7. 'old age'
8. senescence [preferred term]
9. 'virtual realit*'
10. vr
11. 'virtual environment*'
12. immersive
13. audiovisual
14. 'audio visual'
15. 'mixed realit*'
16. interview*
17. 'focus group*'
18. accept*
19. usab*
20. percept*
21. perspect*
22. attitude*
23. feasib*
24. belief*
25. believ*
26. view*
27. barrier*
28. experienc*
29. facilitat*
30. opinion*
31. qualitative
32. 'mixed method*'
33. or/1-8 [sample]
34. or/9-15 [phenomenon of interest]
35. or/16-17 [design]
36. or/18-30 [evaluation]
37. or/31-32 [research type]
38. 33 and 34 and 35 and 36 and 37 [combined search]

Link to open science framework repository: <https://osf.io/ehzg2/>
