# Supplementary material for: Older Adults’ Experiences and Perceptions of Immersive Virtual Reality: Systematic Review and Thematic Synthesis
Source: JMIR Serious Games. 2022 Dec 6;10(4):e35802. doi: 10.2196/35802 (PMC9768659; doi:10.2196/35802)
Supplement: Multimedia Appendix 2 [file games_v10i4e35802_app2.docx]

**Data Extraction Protocol**

Data Extraction

- A data extraction form will be used to extract the data.
  - This will be completed in Excel.
- All data relevant to the items in the data extraction form will be extracted from each study and stored under the relevant item heading.
- Once both reviewers have completed data extraction, they will send their completed data extraction forms to each other for cross-examination.
- A colour-coded system will be used to compare each other’s forms in Excel.
  - Green will be used to confirm that the same data has been extracted in both forms for each item of each study.
  - Yellow will be used to indicate if it is uncertain that the same data has been extracted in both forms for each item of each study.
  - Red will be used to indicate that different data has been extracted in both forms for each item of each study.
- Data Extraction Form Items
  - citation
  - country of publication
  - study setting
  - study design
  - study aims and/or objectives
  - recruitment strategy
  - sample size
  - participant characteristics
    - age
    - sex
    - place of residence
    - mental status
    - work status
    - health status
    - technology experience
  - immersive virtual reality hardware and software systems
  - data analysis technique
  - all data (quotes, themes and author interpretations) reported in the results section relevant to the review question

Quality Appraisal

- Quality appraisal will be completed for all included studies of the review.
- Both reviewers will use The Critical Appraisal Skills Programme (CASP) Qualitative Checklist to assess quality appraisal.
- Once quality appraisal has been completed by both reviewers, any conflicts will be resolved prior to data analysis.

Data Analysis

- Data analysis will be completed by one reviewer.
- On completion of the analysis, the results will be discussed by the entire research team to ensure there is consensus regarding the interpretations made by the reviewer who conducted the analysis.
- Iterations to these interpretations can be made based on the feedback of the other members of the review team at this point.
