# Supplementary material for: Older Adults’ Experiences and Perceptions of Immersive Virtual Reality: Systematic Review and Thematic Synthesis
Source: JMIR Serious Games. 2022 Dec 6;10(4):e35802. doi: 10.2196/35802 (PMC9768659; doi:10.2196/35802)
Supplement: Multimedia Appendix 3 [file games_v10i4e35802_app3.docx]

**Reflective Thematic Analysis Mapped onto Thematic Synthesis**

| **Stage of Thematic Synthesis** | **Steps of Reflexive Thematic Analysis** | **Explanation** |
| --- | --- | --- |
| Initial coding | Familiarisation | This phase first involves familiarising oneself with the data that has been extracted from the studies included in the synthesis. From there, mostly data-derived codes are generated in order to stay close to the findings reported in each study. |
|  | Generating codes (a) |  |
| Descriptive themes | Generating codes (b) | The second phase of Braun and Clarkes’ code generation step best describes the development of descriptive themes, as described by Thomas and Harden. Staying close to the data, similar codes are gathered together into clusters to represent a pattern in the data. Higher-level interpretations are not yet made at this stage. |
| Analytical themes | Constructing themes | Once descriptive themes have been established, higher-level interpretations of these themes can be made. Drawing on patterns from across each of the descriptive themes, a story about the participants’ experiences can begin to form through analytical themes. These are refined until key findings are formed in each analytical theme. These themes are then written up into a narrative in the results section. |
|  | Revising themes |  |
|  | Defining themes |  |
|  | Producing the report |  |
