# Supplementary material for: Older Adults’ Experiences and Perceptions of Immersive Virtual Reality: Systematic Review and Thematic Synthesis
Source: JMIR Serious Games. 2022 Dec 6;10(4):e35802. doi: 10.2196/35802 (PMC9768659; doi:10.2196/35802)
Supplement: Multimedia Appendix 4 [file games_v10i4e35802_app4.docx]

| **Application** | **Description** |
| --- | --- |
| First Contact [15] | - Interactive VR game and tutorial guiding participants through the different features of the hand-held controllers. - Participants are transported to a futuristic lab-like room where they interact with a friendly robot and take part in various tasks including grabbing disk drives and placing them into a 3D printer which generates new objects. - “Requires a moderate to high level of dexterity and mobility” [15]. |
| Quill [15] | - VR animation and illustration tool. - “User draws in a 3D environment using a variety of brushes/pens/colors and other tools. The user can ‘grab’ the drawing and move it in 3D space. The user can also move around the drawing by walking around. There are also pre-programmed ‘tours’ of 3D artwork by famous or experienced artists” [15]. - “Requires a high degree of dexterity and mobility” [15]. |
| Google Earth VR [15,45] | - Enables participants to walk around at street level or fly over places around the world. - “Requires a moderate amount of dexterity and mobility at street-view; Minimal dexterity and mobility for pre-programmed tours” [15]. |
| Ocean Rift [15] | - Described as a VR aquatic safari park. - Divided into 12 habitats which participants can explore and learn about. - “Requires minimal hand dexterity and mobility” [15]. |
| Toybox [15] | - Social interaction game. - Participants can take part in a variety of activities (light fireworks, drive tanks, etc.) with other friends. - “Requires high degree of hand dexterity and mobility” [15]. |
| Power Solitaire [15] | - VR card game. - Use the hand-held controllers to play one or three card solitaire against the computer. - “Requires a moderate degree of hand dexterity and minimal mobility” [15]. |
| Virtual social environment run in the Unity game engine [14,53] | - “The virtual environment depicted in the prototype consisted of an island where the participants’ avatars were seated around a reflective pond that allowed them to look down and see themselves. Audio headsets enabled conversation between the participants via a 3D positional audio Unity plugin” [14]. |
| A series of 6 360-degree videos filmed using a Gear 360 camera [44] | - “The videos consisted of walks (or walk-throughs) and drives around town. This included venues or environments such as a local art museum, a hike on a well-known trail on the edge of town, and an empty church sanctuary. It also included scenes that were filmed while riding as a passenger in a car. (The camera was held outside and above the car by the passenger.) These videos included slowly driving through an old and well-known local neighborhood, the main street through town, and a scenic drive around the grounds of a historic landmark” [44]. |
| Four virtual environments developed using Unity 3D [53,9] | - “The four VEs were designed by a set of governing guidelines for a structured approach to the recreational, nature-based experience of a trail-based exploration in VR. The VEs were designed to complement both a screen-based and HMD configuration, to advocate exploration with a statically forward-facing camera, but with sufficient content for e.g. vertical exploration when wearing a HMD” [9]. |
| 3D VR environment developed using Steam VR tutorial [45,54] | - “The demonstration incorporated the various controlling buttons, trackpads, and visual elements of the equipment and lasted for approximately 4 minutes” [45]. |
| Exercise program developed using Unity 3D [53,46] | - “The ACG system included four mini-games to deliver exercise tasks based on four exercises… knee bends, the user bends their knees to duck under passing logs… leg abduction, the user raises their leg to strike balls positioned to the side… sideways walking, the user steps to the side to avoid oncoming walls… one leg stand, the user stands on one leg to avoid rising water…” |
| The Blu [47] | - Enables participants to explore and interact with different habitats in the ocean. |
| Fruit Ninja [47] | - Activity game where participants cut up fruit for points. |
| Tilt Brush [47] | - 3D VR canvas which affords participants the opportunity to learn how to draw. |
| VR China (developed by National Geographic, China) [16] | - This is an 8-minute long 360-degree video containing sceneries from major natural and cultural attractions in China. |
| Wanna dance? [48] | - “…the player is invited to dance. In this game, the graphical perspective is rendered from the point of view of the user’s character”. |
| The V-Armchair [49] | - “The V-Armchair is a 4D cinematic VR experience based around the simultaneous recording of 360 video and telematics, on a wooden roller coaster. The video is played back on an Oculus Rift while the telematics are played back via a motion-controlled chair, essentially a 6 Degree of Freedom (DOF) Stewart Platform”. |
| Jurassic World [50] | - Audio-visual VR experience displaying scenes of the Jurassic period. |
| Cirque du Soleil [50] | - Audio-visual VR experience displaying scenes of the Cirque du Soleil. |
| 360-degree Virtual Reality Travel Media for Elderly [51] | - 360-degree travel application displaying different places to participants using VR. |
| Finding Wonderland [52] | - “Game Story: With the fairy tale Little Red Riding Hood as the background of the game story, the player will play the role of helping Little Red Riding Hood in the game. After passing various levels, the player will lead the Red Riding Hood who lost in the forest to reach the grandmother's home” [52]. |
