# Supplementary material for: Older Adults’ Experiences and Perceptions of Immersive Virtual Reality: Systematic Review and Thematic Synthesis
Source: JMIR Serious Games. 2022 Dec 6;10(4):e35802. doi: 10.2196/35802 (PMC9768659; doi:10.2196/35802)
Supplement: Multimedia Appendix 5 [file games_v10i4e35802_app5.docx]

| Activities | Description | Quotes |
| --- | --- | --- |
|  |  |  |
| **Travel and exploration** [15,16,44,45,47,50,51] | There was great interest in the ability to travel to places around the world in IVR, suggesting there is utility in IVR to enhance older adults’ travel experiences, particularly to places of significant value to them. Participants from some studies also saw the utility of IVR specifically for people who cannot leave a certain place, with this technology affording them the opportunity to go anywhere without leaving their chair [15,16,50]. | “I can watch it at home. I do not need to go outside and spend money” [16] |
|  |  | “*Participants strongly connected with the authenticity of the VR experience, in combination with a strong acceptance of the Google Earth maps in terms of utility and authenticity*” [45] |
|  |  | “It is suitable for the elderly who can't go out of the door” [16] |
| **Social connection** [14,15,44,48,50,52] | Reported social networking activities for IVR were wide-ranging, including playing games with other people and attending family events through IVR [15,44]. Participants also showed preference to connecting with others through games in a more interactive way [15]. Some negative experiences were also apparent during social networking activities [14]. | “[attend] weddings, birthdays, and sports events in which a grandchild may be playing” [44] |
|  |  | “*she [a participant] would like to combine the interaction from First Contact [an interactive Oculus game] with the skill of Power Solitaire [a card game in virtual reality]*” [15] |
|  |  | “As you get older, you’re less mobile and you can see...you can bring people together in [social VR]...you’d [feel] ‘I’m not going to get on the bus...but let’s get together in [social VR]’” [14] |
|  |  | “*With regard to body tracking technology, while tracking errors of differing severity impact on the usability of all commercially available VR systems, our findings highlight how damaging tracking errors can be in social VR systems where users are looking to convey social meaning via non-verbal cues*” [14] |
| **Entertainment** [15,47,49,50] | IVR increased entertainment options for people living in residential aged care facilities [15,50]. This was expressed in the context of the different applications used by participants during their IVR experience–with many participants enjoying the new forms of entertainment afforded to them by this novel technology [15,50], while others found it did not compare to real life [15]. | “*she felt the software was not ideal as it was difficult to navigate around a 3D painting while in a wheelchair*” [15] |
|  |  | “If Santa brought me one, and it was movies you could get, this might be an excellent way” [50] |
|  |  | “*Nancy said that VR was* ‘much more appealing’ *to her than the currently available activities because* ‘it's just hard to find somebody here that can do, and talk and...play cards with. They all fall asleep.’ *She said that an advantage of VR was that she could interact with the content in ways that "you don't get from everybody [in the residential aged care facility] to the level you want*” [15] |
| **Exercise** [9,46,48] | IVR activities used for exercise included dance [48], a strength and balance exercise program [46], and cycling in a scenic landscape [9]. Some reports indicated this mode of delivery was acceptable [46], while others considered it unfeasible without assistance [9]. | “I relaxed, it was relaxing you dance there together, participate, because everything you like to do, you feel motivated to do, you do not tire” [48] |
|  |  | “*Some participants took breaks from the biking and used them to look around the VE [virtual environment]… Breaks appeared to facilitate a more in-depth impression of the VE content and allow the participants to have longer sessions*” [9] |
|  |  | “When you look around [the virtual environment], you forget that you are exercising. You just follow what is going on” [9] |
|  |  | “*For its usability with the exercise routine intervened in this study, the HMD is not feasible. For manuped exercise purposes, an HMD such as the Oculus DK2 requires its cabinet’s volume in free space in front of it, which in one instance almost became a real issue, as some residents unknowingly only just missed the HMD cabinet for each hand-pedal cycle*” [9] |
| **Education** [14,47,50] | Participants were keen to learn about various topics through IVR, specifically taking advantage of the immersive nature of the technology [50]. Another suggestion made by one participant was to use IVR as a perspective taking tool for healthcare professionals [14]. | “You could find out what it would be like to fly a plane, but I would like to do it myself, you know. That kind of interaction” [50] |
|  |  | “*Samuel… believed that avatars could provide a means for health care professionals to gain a better understanding of what it was like to be an older person and this experience might lead to them* ‘treat[ing] their patients a lot differently’” [14] |
|  |  | “Well I always thought, when studying history, that it would be absolutely wonderful to have a holographic room–with images and with people and with smells and with sounds and you walk into it and you’re in Plymouth in 1622. This is a step in that direction” [50] |
| **Reminiscence** [44,48–50] | Familiar content in IVR afforded participants the opportunity to reminisce on past experiences [44,48–50]. Experiences like this brought some participants comfort [48]. Reminiscence in IVR enables participants to enjoy past memories in a more meaningful and engaged way, reconnecting with an experience that is now missing in their lives [50]. This can also cause feelings of anxiety or depression, however, as it may highlight activities that participants can no longer perform in reality [44]. | “*…while viewing the video of walking along a trail, one female told the team about her and her spouse walking the same trail years ago and how he would conduct fieldwork [plant research] on a nearby hill that could be seen in the video*” [44] |
|  |  | “Comforted, the same as if I were inside my house” [48] |
|  |  | “Seeing the things you have seen before… Oh, I enjoy it and you recall if you’re really interested in that type of thing, the people that were with you and, and the whole occasion and that helps when you can’t go out now” [50] |
