# Supplementary material for: Older Adults’ Experiences and Perceptions of Immersive Virtual Reality: Systematic Review and Thematic Synthesis
Source: JMIR Serious Games. 2022 Dec 6;10(4):e35802. doi: 10.2196/35802 (PMC9768659; doi:10.2196/35802)
Supplement: Multimedia Appendix 6 [file games_v10i4e35802_app6.docx]

|  | Was there a clear statement of the aims of the research? | Is a qualitative methodology appropriate? | Was the research design appropriate to address the aims of the research? | Was the recruitment strategy appropriate to the aims of the research? | Was the data collected in a way that addressed the research issue? | Has the relationship between researcher and participants been adequately considered? | Have ethical issues been taken into consideration? | Was the data analysis sufficiently rigorous? | Is there a clear statement of findings? |
| --- | --- | --- | --- | --- | --- | --- | --- | --- | --- |
| Baker et al. (2020) | Yes | Yes | Yes | Yes | Yes | Can't Tell | Yes | Yes | Yes |
| Liu et al,. (2020) | Yes | Yes | Yes | Yes | Yes | Can't Tell | Can't Tell | Yes | Yes |
| Baker et al. (2019) | Yes | Yes | Yes | Yes | Yes | Can't Tell | Yes | Yes | Yes |
| Brown (2019) | Yes | Yes | Yes | Yes | Yes | Can't Tell | Yes | Yes | Yes |
| Bruun-Pedersen et al. (2016) | Yes | Can't Tell | Can't Tell | Yes | Yes | Can't Tell | No | Yes | Can't Tell |
| Coldham and Cook (2017) | Yes | Yes | Yes | Can't Tell | Can't Tell | Can't Tell | No | Can't Tell | Yes |
| Howes et al. (2019) | Yes | Yes | Yes | Yes | Yes | Can't Tell | Yes | No | Yes |
| Lai et al. (2019) | Yes | Can't Tell | Can't Tell | Can't Tell | Can't Tell | No | No | No | Can't Tell |
| Mol et al. (2019) | Yes | Yes | Yes | Can't Tell | Yes | Can't Tell | Yes | Yes | Yes |
| Passmore et al. (2017) | Can't Tell | Yes | Can't Tell | Can't Tell | Yes | No | Can't Tell | Yes | Yes |
| Roberts et al. (2019) | Yes | Yes | Yes | Yes | Yes | Can't Tell | Can't Tell | Yes | Yes |
| Yang (2019) | Yes | Can't Tell | Can't Tell | Can't Tell | Can't Tell | No | No | No | Can't Tell |
| Srifar (2018)^a^ | Yes | Can't Tell | Can't Tell | Can't Tell | Can't Tell | No | No | No | Can't Tell |

^a^This paper was still under review at the time of analysis. This went unrecognised by the authors until after the review was completed and submitted. It is being noted here for transparency.
