# Supplementary material for: Older Adults’ Experiences and Perceptions of Immersive Virtual Reality: Systematic Review and Thematic Synthesis
Source: JMIR Serious Games. 2022 Dec 6;10(4):e35802. doi: 10.2196/35802 (PMC9768659; doi:10.2196/35802)
Supplement: Multimedia Appendix 7 [file games_v10i4e35802_app7.docx]

| **Summary of review finding** | **Studies contributing to the review finding** | **Methodological limitations** | **Coherence** | **Adequacy** | **Relevance** | **CERQual assessment of confidence in the evidence** | **Explanation of CERQual assessment** |
| --- | --- | --- | --- | --- | --- | --- | --- |
| **Practical aspects of IVR use** |  |  |  |  |  |  |  |
| Finding 1: Interacting with IVR hardware and software | Baker et al. (2019)  Baker et al. (2020)  Brown (2019)  Bruun-Pedersen et al. (2016)  Coldham and Cook (2017)  Howes et al. (2019)  Lai et al. (2019)  Liu et al. (2020)  Mol et al. (2019)  Passmore et al. (2017)  Roberts et al. (2019)  Srifar (2018)  Yang (2019) | *Moderate concerns*  Moderate concerns regarding methodological limitations were determined due uncertainty as to whether ethical issues were taken into consideration in 3 studies and no ethical issues being taken into consideration in 5 of the studies. Uncertainty around researcher reflexivity towards their relationship with the research participants was also observed in 9 of the studies and no consideration for researcher reflexivity in 4 of the studies. | *No or very minor concerns*  This finding was descriptive in nature, meaning the data used to generate it can be clearly compared for its coherence. The data mostly included reports of participants’ experiences interacting with IVR. There was a coherent thread throughout the data used to inform | *No or very minor concerns*  Each study adequately explored older adults’ interactions IVR which all contributed to the forming of this finding. This finding can be described as descriptive, with each of the contributing studies adequately informing the finding at a descriptive level, meaning there are no or very minor concerns about data adequacy. | *No or very minor concerns*  No or very minor concerns regarding relevance. A mix of countries (9), continents (5) and settings. | **Moderate confidence** | 13 studies contributed to this review finding. Moderate concerns regarding methodological limitations. No or very minor concerns regarding coherence, adequacy and relevance. |
| Finding 2: Risks and requirements of IVR use | Baker et al. (2019)  Baker et al. (2020)  Brown (2019)  Bruun-Pedersen et al. (2016)  Coldham and Cook (2017)  Howes et al. (2019)  Lai et al. (2019)  Liu et al. (2020)  Mol et al. (2019)  Roberts et al. (2019)  Srifar (2018) | *No or very minor concerns*  No or very minor concerns regarding methodological limitations were determined due to reliable ratings across the CASP tool in the main studies informing this finding. | *No or very minor concerns*  It was clear that the data informing this finding was in coherence with it as each of the barriers to IVR facilitation that made up this finding were drawn directly from the data. | *No or very minor concerns*  Each study adequately explored the barriers to facilitating IVR use for older adults which all contributed to the forming of this finding. This finding can be described as descriptive, with each of the contributing studies adequately informing the finding at a descriptive level, meaning there are no or very minor concerns about data adequacy. | *No or very minor concerns*  No or very minor concerns regarding relevance. A mix of countries (7), continents (5) and settings. | **High confidence** | 11 studies contributed to this review finding. No or very minor concerns regarding methodological limitations, coherence, adequacy and relevance. |
| **Experiencing unique features of IVR** |  |  |  |  |  |  |  |
| Finding 3: Presence and immersion | Baker et al. (2019)  Baker et al. (2020)  Bruun-Pedersen et al. (2016)  Howes et al. (2019)  Liu et al. (2020)  Mol et al. (2019)  Passmore et al. (2017)  Roberts et al. (2019)  Srifar (2018)  Yang (2019) | *Minor concerns*  Minor concerns regarding methodological limitations were determined due uncertainty as to whether ethical issues were taken into consideration in 3 studies and no ethical issues being taken into consideration in 5 of the studies. Uncertainty around researcher reflexivity towards their relationship with the research participants was also observed in 7 of the studies and no consideration for researcher reflexivity in 3 of the studies. | *No or very minor concerns*  The data informing this finding is in coherence with it, with the data mostly consisting of reports of participants’ experiences with presence and/or immersion. | *No or very minor concerns*  Each study adequately explored older adults’ experience of presence and immersion in IVR which all contributed to the forming of this finding. This finding can be described as descriptive, with each of the contributing studies adequately informing the finding at a descriptive level, meaning there are no or very minor concerns about data adequacy. | *No or very minor concerns*  No or very minor concerns regarding relevance. A mix of countries (9), continents (5) and settings. | **High confidence** | 10 studies contributed to this review finding. Minor concerns regarding methodological limitations. No or very minor concerns regarding coherence, adequacy and relevance. |
| Finding 4: Emotions experienced in IVR | Baker et al. (2019)  Baker et al. (2020)  Brown (2019)  Bruun-Pedersen et al. (2016)  Coldham and Cook (2017)  Howes et al. (2019)  Lai et al. (2019)  Liu et al. (2020)  Mol et al. (2019)  Passmore et al. (2017)  Roberts et al. (2019)  Srifar (2018)  Yang (2019) | *Moderate concerns*  Moderate concerns regarding methodological limitations were determined due uncertainty as to whether ethical issues were taken into consideration in 3 studies and no ethical issues being taken into consideration in 3 of the studies. The 3 studies who were considered to have taken no ethical issues into consideration were deemed to have contributed to this finding in a very minor way, however. Uncertainty around researcher reflexivity towards their relationship with the research participants was also observed in 9 of the studies and no consideration for researcher reflexivity in 4 of the studies. | *Minor concerns*  Although much of the data informing this finding includes some form of sensation or emotional experience with IVR, there are some instances where these experiences do not directly align with the review finding. | *No or very minor concerns*  Each study adequately explored the sensations and emotions older adults experienced in IVR which all contributed to the forming of this finding. This finding can be described as descriptive, with each of the contributing studies adequately informing the finding at a descriptive level, meaning there are no or very minor concerns about data adequacy. | *No or very minor concerns*  No or very minor concerns regarding relevance. A mix of countries (9), continents (5) and settings. | **Moderate confidence** | 13 studies contributed to this review finding. Moderate concerns regarding methodological limitations. Minor concerns regarding coherence. No or very minor concerns regarding adequacy and relevance. |
| Finding 5: Embodied experiences | Baker et al. (2019)  Baker et al. (2020)  Brown (2019)  Passmore et al. (2017) | *No or very minor concerns*  No or very minor concerns regarding methodological limitations were determined due to the mostly reliable ratings across the CASP tool in the studies informing this finding. One study was assessed as having moderate concerns regarding methodological limitations. However, this study did not contribute to this finding in a way that was considered significant enough to impact the overall assessment of its methodological limitations. | *No or very minor concerns*  Coherence between the data informing the finding and the finding itself as the data report only on experiences participants had with how they were represented in the virtual environment. | *Moderate concerns*  Each study adequately explored older adults’ experience of embodiment in IVR which all contributed to the forming of this finding. This finding can be described as descriptive, with each of the contributing studies adequately informing the finding at a descriptive level, meaning there are no or very minor concerns about data adequacy.  Small number of studies attributed to this finding. One study provided most of the data. Thin data from 3 of the studies (2 codes). This raised concerns as to the quantity and quality of the data pertaining to this finding. However, the descriptive nature of this study afforded moderate concerns. | *Moderate concerns*  Moderate concerns regarding relevance. A limited number of countries (3), continents (2) and settings. | **Moderate confidence** | 4 studies contributed to this review finding. No or very minor concerns regarding methodological limitations and coherence. Moderate concerns regarding adequacy and relevance. |
| **Perceptions of IVR** |  |  |  |  |  |  |  |
| Finding 6: Preconceptions of IVR | Baker et al. (2020)  Brown (2019)  Coldham and Cook (2017)  Lai et al. (2019) | *Moderate concerns*  Moderate concerns regarding methodological limitations were determined due no ethical issues being taken into consideration in 1 of the main studies informing this finding. Uncertainty around researcher reflexivity towards their relationship with the research participants was also observed in 3 of the studies and no consideration for researcher reflexivity in 1 of the studies. | *No or very minor concerns*  The range of preconceptions of IVR voiced by participants across each of the studies were explored in this review finding, including the positive and negative preconceptions as well as practical concerns. | *Moderate concerns*  Each study adequately explored older adults’ preconceptions of IVR which all contributed to the forming of this finding. However, the richness of this data could be considered thin, meaning there are moderate concerns about data adequacy.  Descriptive theme: one of the studies provided numerous references to the theme. However, thin data provided from other studies. Given the broad nature of the theme, there is limited quantity to support the finding thus, raising moderate concerns. | *Moderate concerns*  Moderate concerns regarding relevance. A limited number of countries (3), continents (3) and settings. | **Moderate confidence** | 4 studies contributed to this review finding. Moderate concerns regarding methodological limitations, adequacy and relevance. No or very minor concerns regarding coherence. |
| Finding 7: Perceptions of IVR after use | Baker et al. (2019)  Baker et al. (2020)  Brown (2019)  Bruun-Pedersen et al. (2016)  Coldham and Cook (2017)  Howes et al. (2019)  Lai et al. (2019)  Liu et al. (2020)  Mol et al. (2019)  Passmore et al. (2017)  Roberts et al. (2019)  Srifar (2018)  Yang (2019) | *Moderate concerns*  Moderate concerns regarding methodological limitations were determined due uncertainty as to whether ethical issues were taken into consideration in 3 studies and no ethical issues being taken into consideration in 5 of the studies. Uncertainty around researcher reflexivity towards their relationship with the research participants was also observed in 9 of the studies and no consideration for researcher reflexivity in 4 of the studies. | *No or very minor concerns*  The range of perceptions of participants’ experience with IVR were captured in this finding, including the positive and negative perceptions of their experience and the reasons for these perceptions. | *No or very minor concerns*  Each study adequately explored older adults’ perceptions of IVR during and after use which all contributed to the forming of this finding. Although the richness of this data could be considered thin, this was taken into consideration when forming this finding initially meaning there is no or very minor concerns about data adequacy.  Descriptive theme with broad scope therefore, quantity of data is sufficient over richness of data. Good quantity of data although thin in places, among included studies relating to older adults’ perceptions of IVR after use. | *No or very minor concerns*  No or very minor concerns regarding relevance. A mix of countries (9), continents (5) and settings. | **Moderate confidence** | 13 studies contributed to this review finding. Moderate concerns regarding methodological limitations. No or very minor concerns regarding coherence, adequacy and relevance. |
| **Finding 8: Tolerating the bad to experience the good** | Baker et al. (2019)  Baker et al. (2020)  Brown (2019)  Bruun-Pedersen et al. (2016)  Coldham and Cook (2017)  Howes et al. (2019)  Lai et al. (2019)  Liu et al. (2020)  Mol et al. (2019)  Passmore et al. (2017)  Roberts et al. (2019)  Srifar (2018)  Yang (2019) | *Moderate concerns*  As it is a combination of findings 1-5, it was determined that minor concerns regarding methodological limitations would be an appropriate assessment as these findings were a combination of no or very minor concerns to moderate concerns. The main studies contributing to this finding were determined to have no or very minor concerns regarding methodological limitations.  No or very minor (1), Minor (1). Moderate (2).  Two largest contributing studies have moderate concerns. | *No or very minor concerns*  This finding combines other findings relating to facilitating IVR interactions and unique experiences with IVR. There were no concerns about the coherence between the data and this finding as the data helps highlight how participants were keen to use IVR for various purposes in spite of the many issues raised about the current state of the technology. | *Minor concerns*  Each study adequately contributed to the forming of this finding. This finding is more interpretive in nature and draws on the descriptive accounts provided in the above findings to inform it. Although each study contributed to this finding, the quantity of data informing reports of tolerability was limited, meaning there are minor concerns about data adequacy. | *No or very minor concerns*  No or very minor concerns regarding relevance. A mix of countries (9), continents (5) and settings. | **Moderate confidence** | 13 studies contributed to this review finding. Moderate concerns regarding methodological limitations. No or very minor concerns regarding coherence and relevance. Minor concerns regarding adequacy. |
| **Finding 9: Buying in to IVR: don’t judge a book by its cover** | Baker et al. (2019)  Baker et al. (2020)  Brown (2019)  Bruun-Pedersen et al. (2016)  Coldham and Cook (2017)  Howes et al. (2019)  Lai et al. (2019)  Liu et al. (2020)  Mol et al. (2019)  Passmore et al. (2017)  Roberts et al. (2019)  Srifar (2018)  Yang (2019) | *Moderate concerns*  As it is a combination of findings 6 and 7, it was determined that moderate concerns regarding methodological limitations would be an appropriate assessment as these findings were both determined to be moderate concerns regarding methodological limitations. | *No or very minor concerns*  The data informing this finding is deemed to be coherent with the finding itself as it clearly illustrates the temporal pattern in participants perceptions of IVR before, during and after use. | *Moderate concerns*  Each study adequately explored older adults’ perceptions of IVR before, during and after use which all contributed to the forming of this finding. However, the richness of this data could be considered thin, meaning there are moderate concerns about data adequacy. | *No or very minor concerns*  No or very minor concerns regarding relevance. A mix of countries (9), continents (5) and settings. | **Moderate confidence** | 13 studies contributed to this review finding. Moderate concerns regarding methodological limitations. No or very minor concerns regarding coherence and relevance. Moderate concerns regarding adequacy. |
| **Finding 10: “It proves to me I can do it”** | Baker et al. (2019)  Baker et al. (2020)  Brown (2019)  Bruun-Pedersen et al. (2016)  Coldham and Cook (2017)  Howes et al. (2019)  Lai et al. (2019)  Liu et al. (2020)  Mol et al. (2019)  Passmore et al. (2017)  Roberts et al. (2019)  Srifar (2018)  Yang (2019) | *No or very minor concerns*  As it is a combination of finding 8, which has minor concerns regarding methodological limitations, and additional data referring to activities taken part in in IVR which were mostly identified in studies with no or very minor concerns regarding methodological limitations, it was determined that minor concerns regarding methodological limitations would be an appropriate assessment. | *No or very minor concerns*  The data informing this finding is considered to be coherent as it is clear through the data alone how exactly agency is achieved through IVR for the participants and what that means for them. | *Minor concerns*  Each study adequately explored older adults’ accounts in IVR as well as how the activities they took part in facilitated this agency which all contributed to the forming of this finding. Although each study contributed to this finding, the quantity of data informing it is limited, meaning there are minor concerns about data adequacy. | *No or very minor concerns*  No or very minor concerns regarding relevance. A mix of countries (9), continents (5) and settings. | **High confidence** | 13 studies contributed to this review finding. No or very minor concerns regarding methodological limitations, coherence, and relevance. Minor concerns regarding adequacy. |
